# 1′,6-Dimethyl-4′-phenyl­dispiro­[1-benzopyran-3(4*H*),3′-pyrrolidine-2′,3′′-indoline]-2,2′′-dione

**DOI:** 10.1107/S1600536811050768

**Published:** 2011-11-30

**Authors:** D. Lakshmanan, S. Murugavel, D. Kannan, M. Bakthadoss

**Affiliations:** a Department of Physics, C. Abdul Hakeem College of Engineering & Technology, Melvisharam, Vellore 632 509, India; b Department of Physics, Thanthai Periyar Government Institute of Technology, Vellore 632 002, India; c Department of Organic Chemistry, University of Madras, Maraimalai Campus, Chennai 600 025, India

## Abstract

In the title compound, C_27_H_24_N_2_O_3_, the five-membered pyrroldine ring adopts an envelope conformation (with the N atom in the flap position) and the six-membered pyran­one ring of the coumarine ring system adopts a slightly distorted boat conformation. The oxindole unit makes dihedral angles of 89.7 (1) and 25.6 (1)°, respectively, with the pyrrolidine ring and the coumarin ring system. The mol­ecular structure is stabilized by two intra­molecular C—H⋯O contacts and two intra­molecular π–π inter­actions [centroid–centroid seperations of 3.514 (1) and 3.623 (1) Å]. The crystal packing features N—H⋯O hydrogen bonds, which link the mol­ecules into cyclic centrosymmetric *R*_2_^2^(8) dimers, and C—H⋯π inter­actions.

## Related literature

For background to the applications of pyrrolidine derivatives, see: Huryn *et al.* (1991 ▶) ; Suzuki *et al.* (1994 ▶); Waldmann (1995 ▶). For ring puckering analysis, see: Cremer & Pople (1975 ▶) . For closely related pyrrolidine structures, see: Selvanayagam *et al.* (2011 ▶); Ali *et al.* (2010 ▶). 
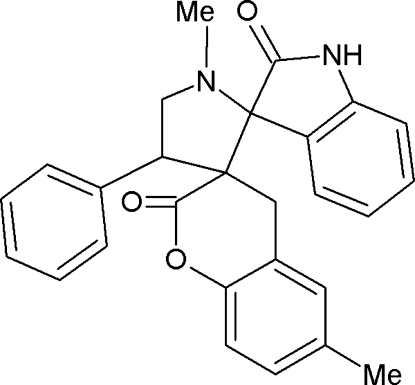


## Experimental

### 

#### Crystal data

C_27_H_24_N_2_O_3_
*M* *_r_* = 424.48
Monoclinic, 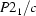

*a* = 11.1019 (5) Å
*b* = 11.1740 (4) Å
*c* = 17.8156 (7) Å
β = 100.986 (2)°
*V* = 2169.57 (15) Å^3^
*Z* = 4
Mo *K*α radiation
μ = 0.09 mm^−1^
*T* = 293 K
0.25 × 0.22 × 0.17 mm

#### Data collection

Bruker APEXII CCD diffractometer
Absorption correction: multi-scan (*SADABS*; Sheldrick, 1996 ▶) *T*_min_ = 0.979, *T*_max_ = 0.986
20613 measured reflections
4481 independent reflections
3019 reflections with *I* > 2σ(*I*)
*R*_int_ = 0.033

#### Refinement

*R*[*F* ^2^ > 2σ(*F* ^2^)] = 0.043
*wR*(*F* ^2^) = 0.116
*S* = 1.02
4481 reflections
291 parameters
H-atom parameters constrained
Δρ_max_ = 0.17 e Å^−3^
Δρ_min_ = −0.18 e Å^−3^

### 

Data collection: *APEX2* (Bruker, 2004 ▶); cell refinement: *APEX2* and *SAINT* (Bruker, 2004 ▶); data reduction: *SAINT* and *XPREP* (Bruker, 2004 ▶); program(s) used to solve structure: *SHELXS97* (Sheldrick, 2008 ▶); program(s) used to refine structure: *SHELXL97* (Sheldrick, 2008 ▶); molecular graphics: *ORTEP-3* (Farrugia (1997 ▶); software used to prepare material for publication: *SHELXL97* and *PLATON* (Spek, 2009 ▶).

## Supplementary Material

Crystal structure: contains datablock(s) global, I. DOI: 10.1107/S1600536811050768/bt5722sup1.cif

Structure factors: contains datablock(s) I. DOI: 10.1107/S1600536811050768/bt5722Isup2.hkl

Additional supplementary materials:  crystallographic information; 3D view; checkCIF report

## Figures and Tables

**Table 1:** Hydrogen-bond geometry (Å, °) *Cg*2 is the centroid of the C7–C12 benzene ring.

*D*—H⋯*A*	*D*—H	H⋯*A*	*D*⋯*A*	*D*—H⋯*A*
C13—H13*A*⋯O1	0.97	2.44	3.114 (2)	126
C26—H26⋯O1	0.93	2.48	3.265 (2)	142
N2—H2⋯O1^i^	0.86	2.06	2.852 (2)	153
C18—H18⋯*Cg*2^ii^	0.93	2.88	3.758 (2)	157

Symmetry codes: (i) 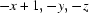; (ii) 
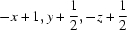
.

**Fig. 1.**
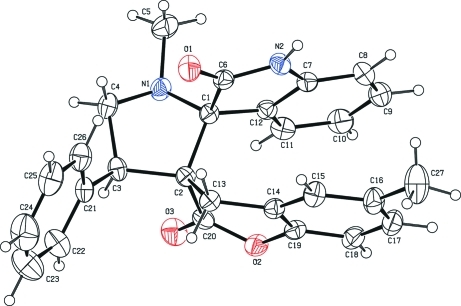
The molecular structure of the title compound with the atom numbering scheme. Displacement ellipsoids are drawn at the 30% probability level.

**Fig. 2.**
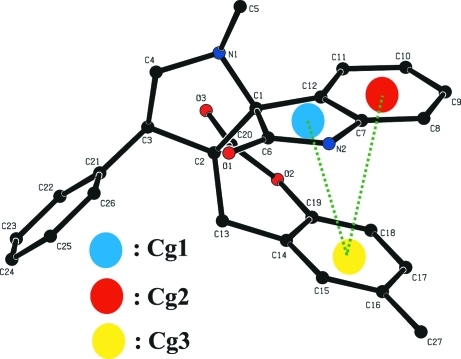
A view of the π—π interactions (dotted lines) in the molecular structure of the title compound. Cg1, Cg2 and Cg3 are the centroids of the (N2/C1/C6/C7/C12) indole ring, (C7–C12) benzene ring and (C14–C19) benzene ring, respectively

**Fig. 3.**
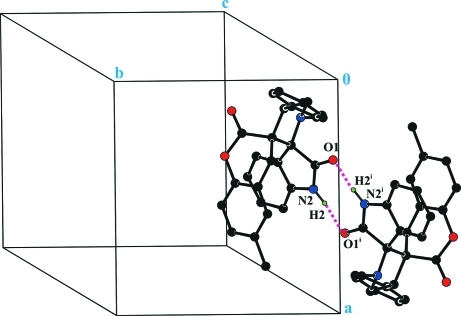
Part of the crystal structure of the title compound showing N—H···O intermolecular hydrogen bonds (dotted lines) generating R22(8) centrosymmetric dimer. [Symmetry code: (i) 1-x, -y, -z].

**Fig. 4.**
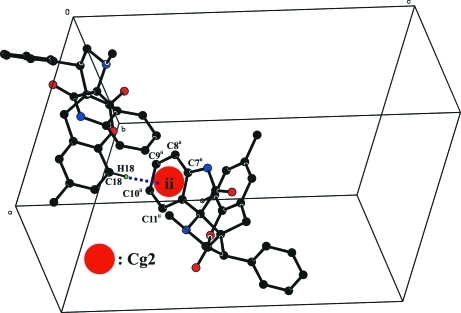
Part of the crystal structure showing C—H···π interactions in the title compound. Cg2 denotes the centroid of the C7–C12 benzene ring. [Symmetry code: (ii) 1-x, 1/2+y, 1/2-z].

## supplementary crystallographic information

### Comment

Highly functionalized pyrrolidines have gained much interest in the past few years as they constitute the main structural element of many natural and synthetic pharmacologically active compounds (Waldmann, 1995). Optically active pyrrolidines have been used as intermediates, chiral ligands or auxiliaries in controlled asymmetric synthesis (Suzuki *et al.,* 1994; Huryn *et al.,* 1991). In view of this importance, the crystal structure of the title compound has been carried out and the results are presented here.

The title compound consists of a pyrrolidine ring connected to a oxindole ring system at C1, a coumarine moiety at C2 and a benzene ring at C3. The X-ray analysis confirms the molecular structure and atom connectivity as illustrated in Fig.1.

The pyrrolidine (N1/C1–C4) ring adopts an envelope conformation with the N1 (displacement = 0.7 (2) Å) atom as the flap atom and with puckering parameters (Cremer & Pople, 1975),q_2_ = 0.3935 (17) Å and φ_2_ = 181.8 (2)°. The six membered pyranone ring (O2/C2/C13/C14/C19/C20) of the coumarine moiety adopts screw-boat conformation as indicated from the puckering parameters: Q = 0.5042 (16) Å, θ = 69.5 (2)° and φ = 208.3 (2)°. The oxindole unit (N2/C1/C6–C12) is essentially planar [maximum deviation = 0.049 (2) Å for the C1 atom] and is oriented at a dihedral angles of 89.7 (1)° and 25.6 (1)°, respectively, with the pyrrolidine and coumarine rings. The sum of angles at N1 of the pyrrolidine ring (336°) is in accordance with *sp*^3^ hybridization, and the sum of angles at N2 of the indole moiety (360°) is in accordance with *sp*^2^ hybridization. The geometric parameters of the title molecule agrees well with those reported for similar structures (Selvanayagam *et al.,* 2011, Ali *et al.,* 2010).

Ihe molecular structure is stabilized by four intramolecular C—H···O contacts (Table 1). The molecular structure is further stabilized by intramolecular π—π interactions with Cg1—Cg3 and Cg2—Cg3 seperations of 3.513 (1) Å and 3.623 (1) Å, respectively (Fig. 2; Cg1, Cg2 and Cg3 are the centroids of the (N2/C1/C6/C7/C12) indole ring, (C7–C12) benzene ring and (C14–C19) benzene ring, respectively). The crystal packing is stabilized by intermolecular N—H···O hydrogen bonds. The molecules at *x, y, z* and *1-x, -y, -z* are linked by N2—H2···O1 hydrogen bonds into cyclic centrosymmetric *R_2_^2^*(8) dimers (Fig. 3). The crystal packing (Fig. 4) is further stabilized by C—H···π interactions between a H18 atom and a neighbouring benzene ring (C7–C12), with a C18—H8···Cg2^ii^ seperation of 2.88 Å ( Fig. 4 and Table 1; Cg2 is the centroid of the C7–C12 benzene ring, Symmetry code as in Fig. 4).

### Experimental

A mixture of *E*-3-benzylidene-6-methylchroman-2-one (0.125 g, 0.5 mmol), isatin (0.08 g, 0.55 mmol) and *N*- methylglycine (0.025 g, 0.55 mmol) in toluene (5 ml) as solvent was allowed to reflux for 6 hours. After work up, the crude mass was purified by column chromatography to yield the pure product (0.199 g, 94 % yield). The compound was recrystallized from ethyl acetate solvent. Single crystals suitable for X-ray diffraction were obtained by slow evaporation of a ethylacetate solution at room temperature.

### Refinement

H atoms were positioned geometrically, with N—H = 0.86 Å and and C—H = 0.93–0.97 Å and constrained to ride on their parent atom, with *U*_iso_(H)=1.5*U*_eq_ for methyl H atoms and 1.2*U*_eq_(C) for other H atoms.

### Crystal data

**Table:**

C_27_H_24_N_2_O_3_	*F*(000) = 896
*M**_r_* = 424.48	*D*_x_ = 1.300 Mg m^−^^3^
Monoclinic, *P*2_1_/*c*	Mo *K*α radiation, λ = 0.71073 Å
Hall symbol: -P 2ybc	Cell parameters from 4502 reflections
*a* = 11.1019 (5) Å	θ = 2.2–26.5°
*b* = 11.1740 (4) Å	µ = 0.09 mm^−^^1^
*c* = 17.8156 (7) Å	*T* = 293 K
β = 100.986 (2)°	Block, colourless
*V* = 2169.57 (15) Å^3^	0.25 × 0.22 × 0.17 mm
*Z* = 4	

### Data collection

**Table:**

Bruker APEXII CCD diffractometer	4481 independent reflections
Radiation source: fine-focus sealed tube	3019 reflections with *I* > 2σ(*I*)
graphite	*R*_int_ = 0.033
Detector resolution: 10.0 pixels mm^-1^	θ_max_ = 26.5°, θ_min_ = 2.2°
ω scans	*h* = −13→13
Absorption correction: multi-scan (*SADABS*; Sheldrick, 1996)	*k* = −9→14
*T*_min_ = 0.979, *T*_max_ = 0.986	*l* = −22→22
20613 measured reflections	

### Refinement

**Table:**

Refinement on *F*^2^	Primary atom site location: structure-invariant direct methods
Least-squares matrix: full	Secondary atom site location: difference Fourier map
*R*[*F*^2^ > 2σ(*F*^2^)] = 0.043	Hydrogen site location: inferred from neighbouring sites
*wR*(*F*^2^) = 0.116	H-atom parameters constrained
*S* = 1.02	*w* = 1/[σ^2^(*F*_o_^2^) + (0.0527*P*)^2^ + 0.3552*P*] where *P* = (*F*_o_^2^ + 2*F*_c_^2^)/3
4481 reflections	(Δ/σ)_max_ < 0.001
291 parameters	Δρ_max_ = 0.17 e Å^−^^3^
0 restraints	Δρ_min_ = −0.18 e Å^−^^3^

### Special details

**Table:**

Geometry. All esds (except the esd in the dihedral angle between two l.s. planes) are estimated using the full covariance matrix. The cell esds are taken into account individually in the estimation of esds in distances, angles and torsion angles; correlations between esds in cell parameters are only used when they are defined by crystal symmetry. An approximate (isotropic) treatment of cell esds is used for estimating esds involving l.s. planes.
Refinement. Refinement of F^2^ against ALL reflections. The weighted R-factor wR and goodness of fit S are based on F^2^, conventional R-factors R are based on F, with F set to zero for negative F^2^. The threshold expression of F^2^ > 2sigma(F^2^) is used only for calculating R-factors(gt) etc. and is not relevant to the choice of reflections for refinement. R-factors based on F^2^ are statistically about twice as large as those based on F, and R- factors based on ALL data will be even larger.

### Fractional atomic coordinates and isotropic or equivalent isotropic displacement parameters (Å^2^)

**Table:**

	*x*	*y*	*z*	*U*_iso_*/*U*_eq_	
O2	0.35755 (11)	0.44958 (10)	0.11728 (6)	0.0466 (3)	
O1	0.34196 (11)	0.02802 (11)	−0.00536 (7)	0.0528 (3)	
N2	0.49556 (12)	0.06511 (12)	0.09708 (8)	0.0435 (3)	
H2	0.5521	0.0230	0.0830	0.052*	
O3	0.16634 (11)	0.41384 (12)	0.12218 (7)	0.0585 (4)	
C12	0.40194 (14)	0.18543 (14)	0.17156 (8)	0.0363 (4)	
N1	0.19474 (12)	0.10995 (13)	0.11455 (8)	0.0442 (4)	
C2	0.25947 (13)	0.27281 (13)	0.04687 (8)	0.0341 (4)	
C14	0.46468 (15)	0.34523 (14)	0.03236 (9)	0.0379 (4)	
C6	0.38139 (15)	0.07699 (14)	0.05603 (9)	0.0392 (4)	
C19	0.46720 (15)	0.41461 (14)	0.09636 (9)	0.0400 (4)	
C13	0.34156 (14)	0.30656 (15)	−0.00996 (8)	0.0380 (4)	
H13A	0.3506	0.2382	−0.0420	0.046*	
H13B	0.3039	0.3710	−0.0427	0.046*	
C15	0.57578 (16)	0.31341 (16)	0.01419 (10)	0.0473 (4)	
H15	0.5765	0.2665	−0.0289	0.057*	
C20	0.25466 (15)	0.38168 (15)	0.09804 (9)	0.0400 (4)	
C7	0.51132 (15)	0.12974 (14)	0.16579 (9)	0.0394 (4)	
C1	0.30808 (14)	0.16185 (14)	0.09964 (8)	0.0351 (4)	
C3	0.12491 (14)	0.23631 (15)	0.00972 (9)	0.0406 (4)	
H3	0.0707	0.2915	0.0303	0.049*	
C8	0.61422 (17)	0.13835 (16)	0.22169 (11)	0.0522 (5)	
H8	0.6868	0.1002	0.2169	0.063*	
C18	0.57418 (17)	0.45345 (15)	0.14141 (10)	0.0490 (5)	
H18	0.5730	0.5013	0.1840	0.059*	
C26	0.12318 (17)	0.16023 (18)	−0.12513 (10)	0.0553 (5)	
H26	0.1662	0.0924	−0.1050	0.066*	
C4	0.10654 (15)	0.11394 (16)	0.04305 (10)	0.0483 (4)	
H4A	0.0235	0.1054	0.0520	0.058*	
H4B	0.1224	0.0508	0.0089	0.058*	
C21	0.09077 (15)	0.24589 (16)	−0.07623 (10)	0.0441 (4)	
C17	0.68353 (17)	0.41960 (17)	0.12189 (11)	0.0546 (5)	
H17	0.7570	0.4445	0.1521	0.065*	
C11	0.39406 (17)	0.24889 (15)	0.23647 (9)	0.0467 (4)	
H11	0.3203	0.2836	0.2424	0.056*	
C16	0.68632 (16)	0.34950 (18)	0.05845 (11)	0.0533 (5)	
C10	0.4981 (2)	0.26011 (17)	0.29294 (10)	0.0555 (5)	
H10	0.4949	0.3049	0.3365	0.067*	
C5	0.20686 (19)	−0.00762 (19)	0.15049 (12)	0.0687 (6)	
H5A	0.1292	−0.0317	0.1617	0.103*	
H5B	0.2667	−0.0041	0.1971	0.103*	
H5C	0.2326	−0.0646	0.1164	0.103*	
C9	0.60616 (19)	0.20576 (18)	0.28527 (11)	0.0579 (5)	
H9	0.6751	0.2147	0.3237	0.069*	
C22	0.02522 (19)	0.3442 (2)	−0.10834 (11)	0.0631 (6)	
H22	0.0017	0.4023	−0.0767	0.076*	
C25	0.0922 (2)	0.1748 (2)	−0.20316 (12)	0.0705 (6)	
H25	0.1149	0.1169	−0.2353	0.085*	
C24	0.0285 (3)	0.2734 (3)	−0.23376 (13)	0.0901 (8)	
H24	0.0087	0.2833	−0.2865	0.108*	
C27	0.8063 (2)	0.3121 (3)	0.03765 (14)	0.0913 (8)	
H27A	0.8728	0.3512	0.0709	0.137*	
H27B	0.8068	0.3342	−0.0144	0.137*	
H27C	0.8156	0.2269	0.0431	0.137*	
C23	−0.0059 (3)	0.3576 (3)	−0.18622 (14)	0.0911 (8)	
H23	−0.0505	0.4242	−0.2068	0.109*	

### Atomic displacement parameters (Å^2^)

**Table:**

	*U*^11^	*U*^22^	*U*^33^	*U*^12^	*U*^13^	*U*^23^
O2	0.0484 (7)	0.0371 (7)	0.0556 (7)	0.0021 (5)	0.0129 (6)	−0.0114 (5)
O1	0.0457 (7)	0.0542 (8)	0.0574 (8)	0.0055 (6)	0.0067 (6)	−0.0225 (6)
N2	0.0361 (8)	0.0411 (8)	0.0535 (9)	0.0086 (6)	0.0088 (7)	−0.0062 (6)
O3	0.0518 (8)	0.0640 (9)	0.0642 (8)	0.0145 (6)	0.0222 (7)	−0.0155 (6)
C12	0.0379 (9)	0.0360 (9)	0.0360 (8)	−0.0006 (7)	0.0094 (7)	0.0040 (7)
N1	0.0375 (8)	0.0495 (9)	0.0470 (8)	−0.0073 (6)	0.0118 (7)	0.0046 (6)
C2	0.0307 (8)	0.0364 (9)	0.0371 (8)	0.0033 (6)	0.0108 (7)	−0.0018 (6)
C14	0.0389 (9)	0.0398 (10)	0.0365 (9)	−0.0039 (7)	0.0107 (7)	0.0047 (7)
C6	0.0380 (10)	0.0332 (9)	0.0471 (10)	0.0004 (7)	0.0094 (8)	−0.0031 (7)
C19	0.0432 (10)	0.0341 (9)	0.0438 (9)	−0.0028 (7)	0.0114 (8)	0.0024 (7)
C13	0.0389 (9)	0.0403 (10)	0.0365 (9)	−0.0013 (7)	0.0118 (7)	−0.0004 (7)
C15	0.0434 (11)	0.0599 (12)	0.0419 (10)	−0.0063 (8)	0.0168 (8)	−0.0018 (8)
C20	0.0411 (10)	0.0397 (10)	0.0403 (9)	0.0087 (7)	0.0101 (8)	0.0011 (7)
C7	0.0395 (10)	0.0364 (10)	0.0420 (9)	−0.0004 (7)	0.0072 (8)	0.0040 (7)
C1	0.0320 (9)	0.0364 (9)	0.0388 (9)	0.0004 (6)	0.0117 (7)	−0.0023 (6)
C3	0.0291 (8)	0.0520 (11)	0.0418 (9)	0.0052 (7)	0.0097 (7)	−0.0034 (7)
C8	0.0437 (11)	0.0516 (12)	0.0572 (11)	0.0063 (8)	−0.0008 (9)	0.0053 (9)
C18	0.0540 (12)	0.0439 (11)	0.0476 (10)	−0.0103 (8)	0.0060 (9)	−0.0043 (8)
C26	0.0453 (11)	0.0694 (14)	0.0519 (11)	−0.0038 (9)	0.0112 (9)	−0.0117 (9)
C4	0.0328 (9)	0.0585 (12)	0.0554 (11)	−0.0083 (8)	0.0131 (8)	−0.0018 (9)
C21	0.0294 (9)	0.0575 (11)	0.0452 (10)	−0.0029 (7)	0.0064 (7)	−0.0039 (8)
C17	0.0460 (11)	0.0616 (13)	0.0535 (11)	−0.0148 (9)	0.0027 (9)	0.0042 (9)
C11	0.0548 (11)	0.0484 (11)	0.0388 (9)	0.0031 (8)	0.0141 (8)	0.0022 (8)
C16	0.0381 (11)	0.0692 (13)	0.0539 (11)	−0.0077 (9)	0.0121 (9)	0.0057 (9)
C10	0.0736 (14)	0.0548 (12)	0.0356 (9)	−0.0020 (10)	0.0043 (9)	−0.0012 (8)
C5	0.0677 (14)	0.0634 (14)	0.0742 (14)	−0.0184 (11)	0.0119 (11)	0.0209 (11)
C9	0.0619 (13)	0.0571 (13)	0.0472 (11)	−0.0044 (10)	−0.0088 (9)	0.0042 (9)
C22	0.0575 (13)	0.0755 (15)	0.0546 (12)	0.0130 (10)	0.0065 (10)	0.0044 (10)
C25	0.0631 (14)	0.0997 (19)	0.0506 (12)	−0.0200 (13)	0.0159 (11)	−0.0186 (12)
C24	0.0876 (19)	0.135 (3)	0.0435 (13)	−0.0132 (17)	0.0025 (12)	0.0093 (15)
C27	0.0437 (13)	0.141 (2)	0.0926 (18)	−0.0061 (14)	0.0221 (12)	−0.0130 (17)
C23	0.098 (2)	0.108 (2)	0.0617 (15)	0.0225 (16)	0.0007 (14)	0.0198 (14)

### Geometric parameters (Å, °)

**Table:**

O2—C20	1.360 (2)	C8—H8	0.9300
O2—C19	1.395 (2)	C18—C17	1.378 (3)
O1—C6	1.2265 (19)	C18—H18	0.9300
N2—C6	1.344 (2)	C26—C25	1.377 (3)
N2—C7	1.403 (2)	C26—C21	1.387 (2)
N2—H2	0.8600	C26—H26	0.9300
O3—C20	1.1984 (18)	C4—H4A	0.9700
C12—C11	1.373 (2)	C4—H4B	0.9700
C12—C7	1.386 (2)	C21—C22	1.380 (3)
C12—C1	1.513 (2)	C17—C16	1.380 (3)
N1—C4	1.452 (2)	C17—H17	0.9300
N1—C1	1.4551 (19)	C11—C10	1.386 (3)
N1—C5	1.456 (2)	C11—H11	0.9300
C2—C20	1.527 (2)	C16—C27	1.508 (3)
C2—C13	1.533 (2)	C10—C9	1.374 (3)
C2—C3	1.568 (2)	C10—H10	0.9300
C2—C1	1.587 (2)	C5—H5A	0.9600
C14—C19	1.375 (2)	C5—H5B	0.9600
C14—C15	1.380 (2)	C5—H5C	0.9600
C14—C13	1.494 (2)	C9—H9	0.9300
C6—C1	1.551 (2)	C22—C23	1.373 (3)
C19—C18	1.371 (2)	C22—H22	0.9300
C13—H13A	0.9700	C25—C24	1.366 (4)
C13—H13B	0.9700	C25—H25	0.9300
C15—C16	1.386 (2)	C24—C23	1.368 (4)
C15—H15	0.9300	C24—H24	0.9300
C7—C8	1.368 (2)	C27—H27A	0.9600
C3—C21	1.510 (2)	C27—H27B	0.9600
C3—C4	1.520 (2)	C27—H27C	0.9600
C3—H3	0.9800	C23—H23	0.9300
C8—C9	1.377 (3)		
			
C20—O2—C19	120.69 (12)	C19—C18—C17	118.16 (16)
C6—N2—C7	111.83 (13)	C19—C18—H18	120.9
C6—N2—H2	124.1	C17—C18—H18	120.9
C7—N2—H2	124.1	C25—C26—C21	120.5 (2)
C11—C12—C7	119.45 (15)	C25—C26—H26	119.7
C11—C12—C1	131.22 (15)	C21—C26—H26	119.7
C7—C12—C1	109.32 (13)	N1—C4—C3	104.60 (13)
C4—N1—C1	106.84 (12)	N1—C4—H4A	110.8
C4—N1—C5	113.81 (14)	C3—C4—H4A	110.8
C1—N1—C5	115.41 (14)	N1—C4—H4B	110.8
C20—C2—C13	106.93 (13)	C3—C4—H4B	110.8
C20—C2—C3	108.67 (12)	H4A—C4—H4B	108.9
C13—C2—C3	115.07 (12)	C22—C21—C26	117.93 (17)
C20—C2—C1	108.38 (12)	C22—C21—C3	119.18 (16)
C13—C2—C1	113.93 (12)	C26—C21—C3	122.89 (16)
C3—C2—C1	103.64 (12)	C18—C17—C16	121.39 (17)
C19—C14—C15	117.51 (15)	C18—C17—H17	119.3
C19—C14—C13	116.99 (14)	C16—C17—H17	119.3
C15—C14—C13	125.45 (15)	C12—C11—C10	118.68 (17)
O1—C6—N2	125.50 (15)	C12—C11—H11	120.7
O1—C6—C1	125.95 (14)	C10—C11—H11	120.7
N2—C6—C1	108.56 (13)	C17—C16—C15	118.37 (17)
C18—C19—C14	122.86 (16)	C17—C16—C27	121.15 (18)
C18—C19—O2	117.23 (15)	C15—C16—C27	120.48 (18)
C14—C19—O2	119.91 (15)	C9—C10—C11	120.71 (17)
C14—C13—C2	109.87 (12)	C9—C10—H10	119.6
C14—C13—H13A	109.7	C11—C10—H10	119.6
C2—C13—H13A	109.7	N1—C5—H5A	109.5
C14—C13—H13B	109.7	N1—C5—H5B	109.5
C2—C13—H13B	109.7	H5A—C5—H5B	109.5
H13A—C13—H13B	108.2	N1—C5—H5C	109.5
C14—C15—C16	121.71 (17)	H5A—C5—H5C	109.5
C14—C15—H15	119.1	H5B—C5—H5C	109.5
C16—C15—H15	119.1	C10—C9—C8	121.21 (18)
O3—C20—O2	116.60 (15)	C10—C9—H9	119.4
O3—C20—C2	125.26 (16)	C8—C9—H9	119.4
O2—C20—C2	118.14 (13)	C23—C22—C21	121.1 (2)
C8—C7—C12	122.43 (16)	C23—C22—H22	119.4
C8—C7—N2	128.25 (16)	C21—C22—H22	119.4
C12—C7—N2	109.30 (14)	C24—C25—C26	120.6 (2)
N1—C1—C12	113.26 (12)	C24—C25—H25	119.7
N1—C1—C6	113.72 (13)	C26—C25—H25	119.7
C12—C1—C6	100.85 (12)	C25—C24—C23	119.5 (2)
N1—C1—C2	102.19 (12)	C25—C24—H24	120.3
C12—C1—C2	117.85 (12)	C23—C24—H24	120.3
C6—C1—C2	109.42 (12)	C16—C27—H27A	109.5
C21—C3—C4	115.70 (14)	C16—C27—H27B	109.5
C21—C3—C2	116.39 (13)	H27A—C27—H27B	109.5
C4—C3—C2	104.89 (12)	C16—C27—H27C	109.5
C21—C3—H3	106.4	H27A—C27—H27C	109.5
C4—C3—H3	106.4	H27B—C27—H27C	109.5
C2—C3—H3	106.4	C24—C23—C22	120.3 (2)
C7—C8—C9	117.45 (17)	C24—C23—H23	119.8
C7—C8—H8	121.3	C22—C23—H23	119.8
C9—C8—H8	121.3		
			
C7—N2—C6—O1	−178.23 (16)	N2—C6—C1—C2	121.92 (14)
C7—N2—C6—C1	1.28 (18)	C20—C2—C1—N1	90.74 (14)
C15—C14—C19—C18	0.6 (2)	C13—C2—C1—N1	−150.36 (12)
C13—C14—C19—C18	178.27 (15)	C3—C2—C1—N1	−24.59 (14)
C15—C14—C19—O2	179.87 (14)	C20—C2—C1—C12	−34.08 (17)
C13—C14—C19—O2	−2.5 (2)	C13—C2—C1—C12	84.81 (16)
C20—O2—C19—C18	−153.29 (15)	C3—C2—C1—C12	−149.41 (13)
C20—O2—C19—C14	27.4 (2)	C20—C2—C1—C6	−148.42 (13)
C19—C14—C13—C2	−39.92 (19)	C13—C2—C1—C6	−29.53 (17)
C15—C14—C13—C2	137.50 (16)	C3—C2—C1—C6	96.25 (14)
C20—C2—C13—C14	56.40 (16)	C20—C2—C3—C21	116.31 (15)
C3—C2—C13—C14	177.18 (13)	C13—C2—C3—C21	−3.5 (2)
C1—C2—C13—C14	−63.32 (17)	C1—C2—C3—C21	−128.57 (14)
C19—C14—C15—C16	0.0 (3)	C20—C2—C3—C4	−114.43 (14)
C13—C14—C15—C16	−177.36 (16)	C13—C2—C3—C4	125.75 (14)
C19—O2—C20—O3	175.48 (14)	C1—C2—C3—C4	0.70 (15)
C19—O2—C20—C2	−5.5 (2)	C12—C7—C8—C9	−0.2 (3)
C13—C2—C20—O3	143.17 (17)	N2—C7—C8—C9	−178.56 (17)
C3—C2—C20—O3	18.4 (2)	C14—C19—C18—C17	−1.0 (3)
C1—C2—C20—O3	−93.61 (19)	O2—C19—C18—C17	179.80 (15)
C13—C2—C20—O2	−35.73 (18)	C1—N1—C4—C3	−42.03 (16)
C3—C2—C20—O2	−160.51 (14)	C5—N1—C4—C3	−170.61 (14)
C1—C2—C20—O2	87.49 (16)	C21—C3—C4—N1	153.41 (14)
C11—C12—C7—C8	−2.1 (2)	C2—C3—C4—N1	23.74 (16)
C1—C12—C7—C8	178.14 (15)	C25—C26—C21—C22	1.1 (3)
C11—C12—C7—N2	176.58 (14)	C25—C26—C21—C3	−178.44 (17)
C1—C12—C7—N2	−3.23 (18)	C4—C3—C21—C22	136.12 (17)
C6—N2—C7—C8	179.74 (16)	C2—C3—C21—C22	−100.01 (19)
C6—N2—C7—C12	1.21 (19)	C4—C3—C21—C26	−44.4 (2)
C4—N1—C1—C12	169.27 (13)	C2—C3—C21—C26	79.5 (2)
C5—N1—C1—C12	−63.08 (19)	C19—C18—C17—C16	0.6 (3)
C4—N1—C1—C6	−76.35 (16)	C7—C12—C11—C10	3.1 (2)
C5—N1—C1—C6	51.30 (19)	C1—C12—C11—C10	−177.14 (16)
C4—N1—C1—C2	41.45 (15)	C18—C17—C16—C15	0.0 (3)
C5—N1—C1—C2	169.10 (14)	C18—C17—C16—C27	−179.60 (19)
C11—C12—C1—N1	−54.2 (2)	C14—C15—C16—C17	−0.4 (3)
C7—C12—C1—N1	125.58 (14)	C14—C15—C16—C27	179.28 (18)
C11—C12—C1—C6	−176.09 (17)	C12—C11—C10—C9	−2.0 (3)
C7—C12—C1—C6	3.69 (16)	C11—C10—C9—C8	−0.3 (3)
C11—C12—C1—C2	65.0 (2)	C7—C8—C9—C10	1.4 (3)
C7—C12—C1—C2	−115.27 (15)	C26—C21—C22—C23	−0.7 (3)
O1—C6—C1—N1	55.0 (2)	C3—C21—C22—C23	178.8 (2)
N2—C6—C1—N1	−124.53 (14)	C21—C26—C25—C24	−0.3 (3)
O1—C6—C1—C12	176.54 (16)	C26—C25—C24—C23	−0.8 (4)
N2—C6—C1—C12	−2.97 (16)	C25—C24—C23—C22	1.2 (4)
O1—C6—C1—C2	−58.6 (2)	C21—C22—C23—C24	−0.4 (4)

### Hydrogen-bond geometry (Å, °)

**Table:**

Cg2 is the centroid of the C7–C12 benzene ring.

**Table:**

*D*—H···*A*	*D*—H	H···*A*	*D*···*A*	*D*—H···*A*
C3—H3···O3	0.98	2.24	2.795 (2)	115
C4—H4B···O1	0.97	2.51	3.059 (2)	116
C13—H13A···O1	0.97	2.44	3.114 (2)	126
C26—H26···O1	0.93	2.48	3.265 (2)	142
N2—H2···O1^i^	0.86	2.06	2.852 (2)	153.
C18—H18···Cg2^ii^	0.93	2.88	3.758 (2)	157

Symmetry codes:  (i) −*x*+1, −*y*, −*z*; (ii) −*x*+1, *y*+1/2, −*z*+1/2.
